# Gastrointestinal Symptoms in Marfan Syndrome and Hypermobile Ehlers-Danlos Syndrome

**DOI:** 10.1155/2018/4854701

**Published:** 2018-07-29

**Authors:** N. Inayet, J. O. Hayat, A. Kaul, M. Tome, A. Child, A. Poullis

**Affiliations:** ^1^Department of Gastroenterology, St. George's Hospital, London, UK; ^2^Department of Rheumatology, St. George's Hospital, London, UK; ^3^Department of Cardiology, St. George's Hospital, London, UK

## Abstract

**Objective:**

Marfan syndrome (MS) is a multisystem disorder caused by a mutation in FBN1 gene. It shares some phenotypic features with hypermobile Ehlers-Danlos syndrome (EDS) such as joint hypermobility. EDS is a group of inherited heterogenous multisystem disorders characterized by skin hyperextensibility, atrophic scarring, joint hypermobility, and generalized tissue fragility. Hypermobile EDS (hEDS) is thought to be the most common type. Recent studies have suggested an association between connective tissue hypermobility and functional gastrointestinal disorders (FGDs). The aim of this study is to determine the prevalence of gastrointestinal symptoms in patients with Marfan syndrome and hypermobile EDS.

**Method:**

Patients with a diagnosis of either MS or hEDS attending cardiology or rheumatology outpatients at our hospital were asked to complete SF36 RAND and Rome IV Diagnostic questionnaires. Questionnaires were also completed by patients who are members of Marfan Association UK. The same questionnaires were also completed by age- and gender-matched controls attending fracture clinic without existing diagnoses of MS or hEDS.

**Results:**

Data were collected from 45 MS patients (12 males and 33 females, age range 19–41 years, mean 28 years) and 45 hEDS patients (6 males and 39 females, age range 18–32 years, mean 24 years). None had a previous organic gastrointestinal diagnosis. The control group was matched for age and sex (18 males and 72 females, age range 18–45, mean 29 years). Both MS and hEDS groups showed a higher prevalence of abdominal symptoms compared to the control group; however, the hEDS group not only showed a higher prevalence but more frequent and severe symptoms meeting Rome IV criteria for diagnosis of FGIDs. Nearly half of the hEDS patients met the criteria for more than one FGID. The hEDS group also scored lower on quality of life (QOL) scores in comparison to either of the other groups with a mean score of 48.6 as compared to 54.2 in the Marfan group and 78.6 in the control group.

**Conclusion:**

FGIDs are reported in both Marfan syndrome and hypermobile Ehlers-Danlos syndrome but appear to be more common and severe in hEDS. These patients score lower on quality of life scores as well despite hypermobility being a common feature of both conditions. Further work is needed to understand the impact of connective tissue disorders on gastrointestinal symptoms.

## 1. Introduction

Marfan syndrome (MS) is a multisystem disorder caused by a mutation in FBN1 gene [1]. It shares some phenotypic features with hypermobile Ehlers-Danlos syndrome (EDS) such as joint hypermobility [2]. EDS is a group of inherited heterogenous multisystem disorders characterized by skin hyperextensibility, atrophic scarring, joint hypermobility, and generalized tissue fragility [3]. Hypermobile EDS (hEDS) is thought to be the most common type [4]. Recent studies have suggested an association between connective tissue hypermobility and functional gastrointestinal disorders (FGDs) [5–10].

Gastrointestinal symptoms are reported to be common in patients with hypermobility; however, much of the recent interest in this area is focused only on patients with hEDS. No previous study has compared the prevalence of gastrointestinal symptoms in patients with MS and compared to patients with hEDS. The aim of our study is to use standardised definitions for all gastrointestinal symptoms using Diagnostic Rome IV questionnaire as well as look for the overall perception of health and well-being using RAND SF36 questionnaire. We have also investigated the use of the most commonly used medications which affect gastrointestinal function in these groups.

## 2. Method

Patients attending outpatients at St. George's Hospital who had an existing diagnosis of MS or hEDS were asked to complete SF36 RAND and Rome IV Diagnostic questionnaires. Some patients were recruited with the help of Marfan Association UK. All patients completed the questionnaires by themselves, either in the Outpatients Department or at home, and returned them by post.

Controls were recruited from outpatients attending the fracture clinic at St. George's Hospital, one control being recruited for each MS and hEDS patient, matched by gender and age (±10 years). Hypermobility was assessed in these controls using Beighton criteria, and scores of 4 or less were included. All patients completed the questionnaires themselves while attending clinic. No financial or other inducements were given.

All recruited subjects completed the SF36 RAND and Rome IV Diagnostic questionnaires. Additional questions regarding ethnicity, smoking and alcohol use, use of medicines, and other medical conditions were added using previously validated questionnaires. All participants were asked if they had used antisecretory/antacid therapy or laxatives, either regularly or on as required basis in the last six months. The scoring algorithm for Rome IV Diagnostic questionnaire for adults was used, and a functional gastrointestinal disorder was said to be present if the criteria were met. The presence of abdominal pain, constipation, and diarrhoea was investigated separately even if criteria for FGID were not met.

The Rome IV classification was followed for various gastrointestinal symptoms and diagnoses including the following:

1. Oesophageal disorders (functional chest pain, functional heartburn, Globus, and functional dysphagia)
2. Gastroduodenal disorders (functional dyspepsia, belching disorders, nausea and vomiting disorders, and rumination syndrome)
3. Bowel disorders (irritable bowel syndrome (IBS), functional constipation, functional diarrhoea, functional abdominal bloating/distension, and unspecified functional bowel disorder)
4. Centrally mediated abdominal pain syndrome
5. Functional biliary pain
6. Anorectal disorders (faecal incontinence and functional anorectal pain)

Functional chest pain was defined as retrosternal chest pain or discomfort at least weekly with no heartburn or dysphagia. Functional heartburn was defined as burning retrosternal discomfort or pain with no relief from antisecretory therapy. Globus was defined as persistent or intermittent, nonpainful sensation of a lump or foreign body in the throat with the absence of dysphagia or heartburn. Functional dysphagia was defined as sense of solid and/or liquid foods sticking, lodging, or passing abnormally through the oesophagus in the absence of organic disorders.

Functional dyspepsia was defined as postprandial fullness, early satiation, epigastric pain, or burning, severe enough to impact on usual activities or prevent finishing a regular size meal. Functional belching was defined as belching, severe enough to impact on usual activities, from the oesophagus or stomach more than 3 days a week. Nausea and vomiting disorders were defined as weekly nausea or vomiting episodes, severe enough to impact on usual activities. Rumination syndrome was defined as persistent or recurrent regurgitation of recently ingested food into the mouth with subsequent spitting or remastication and swallowing.

IBS was defined as recurrent abdominal pain related to defecation and associated with a change in frequency or form of stool. IBS subtypes (IBS-C, IBS-D, IBS-M, and IBS-U) were classified based on patient's perception of the usual consistency of abnormal stools, according to Bristol stool scale. Functional constipation was defined as bowel opening associated with two or more of straining, type 1-2 (Bristol stool scale) stools, sensation of incomplete evacuation, sensation of anorectal obstruction, manual manoeuvres to facilitate defecation, fewer than three spontaneous stools per week, and absence of loose stools. Functional diarrhoea was defined as the presence of loose, watery stools (type 5-6 Bristol stool scale) without predominant abdominal pain or bloating. Functional abdominal bloating/distension was defined as recurrent bloating or distension at least weekly where bloating predominates all other symptoms. Unspecified functional bowel disorder was defined as the presence of symptoms of abdominal pain, constipation, diarrhoea, or bloating which do not meet criteria for other conditions.

Centrally mediated abdominal pain syndrome was defined as continuous or nearly continuous abdominal pain with no (or occasional) relationship of pain to physiological events such as eating, defecation, or periods. Functional biliary pain was defined as pain in the epigastrium and/or right upper quadrant that builds up to a steady level, lasts 30 minutes or longer, occurring at different intervals, severe enough to interrupt usual activities and not related to bowel movements, posture change, or acid suppression.

Faecal incontinence was defined as recurrent uncontrolled passage of faecal material. Functional anorectal pain was defined as chronic or recurrent rectal pain or aching with episodes lasting 30 minutes or longer.

All participants completed the SF36 Quality of Life questionnaire. Mean scores out of 100 (where 0 was the worst outcome on the scale and 100 was the best) for all groups were calculated for each of the eight domains, that is, physical functioning, role limitations due to physical health, role limitations due to emotional problems, level of energy/fatigue, emotional well-being, social functioning, pain, and general health.

### 2.1. Statistical Analyses

Statistical analyses were performed using the IBM Statistical Package for the Social Science (SPSS) software version 25 (IBM SPSS Inc., Chicago, IL). The Fisher's exact test and Chi-squared test were used for comparing categorical variables. Independent sample *t-*test values were used to compare the mean scores in different modalities of SF36 questionnaire. As multiple comparisons were made, probability values of <0.05 were thought to be statistically significant.

### 2.2. Ethical Considerations

The study protocol was approved by the South West-Central Bristol Research Ethics Committee and NHS Health Research Authority UK.

## 3. Results

90 patients and 90 controls were included in this study. Data was collected from 45 MS patients (12 males and 33 females, age 19–41, mean 28 years) with no organic gastrointestinal diagnosis. Data was collected from 45 hEDS patients (6 males and 39 females, age 18–32, mean 24 years) with no organic gastrointestinal diagnosis.

### 3.1. Gastrointestinal Symptoms in MS/hEDS Patients

Combining the MS and hEDS patients together, these patients were more likely to report a wide range of gastrointestinal symptoms compared to controls (Table 1).

Abdominal pain, bowel symptoms, heartburn, dyspepsia, and dysphagia were all significantly more common in the combined hypermobile group compared with controls. Medication use was also significantly more common in this group.

### 3.2. Gastrointestinal Symptoms in MS

More Marfan patients reported abdominal pain, diarrhoea, and constipation than controls; however, only symptom of abdominal pain was thought to be statistically significant. The use of antisecretory therapy and laxative was also reported in more MS patients than controls, but this did not reach statistical significance (Table 2).

### 3.3. Gastrointestinal Symptoms and Diagnoses in hEDS Patients (Table 3)

All GI symptoms and use of medicines were considerably higher in the hEDS group meeting statistical significance for all three symptoms and use of medicines. FGIDs were also more frequently found in hEDS group meeting statistical significance for functional heartburn, functional dysphagia, functional dyspepsia, irritable bowel syndrome, functional constipation, functional diarrhoea, functional abdominal bloating, and unspecified bowel disorder (Table 3).

### 3.4. Perception of Health and Well-Being

The SF36 Quality of Life questionnaire generates a mean score out of 100 (where 0 was the worst outcome on the scale and 100 was the best) for eight domains: physical functioning, role limitations due to physical health, role limitations due to emotional problems, level of energy/fatigue, emotional well-being, social functioning, pain, and general health. The mean scores across all domains of MS patients were compared with controls showing statistical significance in most domains (Table 4). Similarly, the mean scores of hEDS patients showed statistical significance in almost all domains when compared to controls (Table 5). The hEDS group, however, scored the lowest on all domains of this questionnaire.

## 4. Discussion

This is the first study of its kind showing that connective tissue hypermobile syndromes are associated with a significant number of gastrointestinal symptoms compared to control patients. Despite the many phenotypic similarities, MS and hEDS exhibit different symptom complexes. The burden of functional gastrointestinal symptoms in MS in comparison with hEDS has never been studied and compared. Individuals with hEDS suffer a greater impairment to general well-being than MS patients.

Marfan syndrome is a spectrum of heritable disorders of connective tissue with a high degree of clinical variability. It has an autosomal dominant mode of transmission mainly affecting the connective tissue protein fibrillin. The defect itself has been isolated to the FBN1 gene on chromosome 15, which codes for the connective tissue protein fibrillin [11]. The most serious and common complications of Marfan syndrome are usually cardiovascular, skeletal, pulmonary, and ocular. Aortic root dilatation and dissection [12] are perhaps the most serious manifestations whereas skeletal deformities [13] including thoracolumbar scoliosis, thoracic lordosis, and pectus excavatum can lead to pulmonary problems [14] if left untreated. In addition, ocular problems such as superior dislocation of the lens, retinal detachment, myopia, glaucoma, and cataracts are common findings and can lead to blindness if remain unrecognized [15–17]. The diagnosis of Marfan syndrome is based on family history, the presence of certain clinical features such as aortic root dilatation and ectopia lentis, and genetic testing for FBN1. The revised Ghent criteria for diagnosing Marfan syndrome were published in 2010 [18, 19].

A variety of large surgical publications have highlighted the anatomical or structural complications involving the gastrointestinal tract in patients with Marfan syndrome [20–32]; however, there have not been many studies looking at GI symptoms in these patients.

Ehlers-Danlos syndrome is described as a group of inherited noninflammatory connective tissue disorders primarily caused by a defect in the synthesis of collagen. The syndrome derives its name from clinical case reports presented by 2 physicians: Edvard Ehlers, a Danish dermatologist in 1901, and Henri-Alexandre Danlos, a French physician in 1908. The types of EDS have traditionally been classified according to the Revised Villefranche Nosology presented in 1997 [33].

The current classification of EDS is based on the 2017 international classification of Ehlers-Danlos syndrome which includes the traditional six types as well as other rarer types [34]. All these types share some clinical manifestations such as joint laxity, soft skin, and easy bruising; however, each type is thought to involve a unique defect in connective tissue, although not all the genes responsible for causing EDS have been found. The hypermobile and classical types are thought to be much more common than the other types. Diagnosis is mainly clinical using Beighton criteria for joint hypermobility in the presence of symptoms of skin elasticity and easy bruising. Skin histopathology and molecular genetics can be helpful in diagnosis.

One of the first descriptions of gastrointestinal involvement in EDS was an observational study published in 1969 [35]. However, more recently, several published studies have shown that there is a high prevalence of functional gastrointestinal symptoms in Ehlers-Danlos syndrome whereas more catastrophic gastrointestinal complications remain relatively rare. Structural abnormalities have been reported in up to 20% of individuals and functional gastrointestinal symptoms in up to 75% [10, 36]. A retrospective study from Mayo Clinic including all Ehlers-Danlos syndrome subtypes reported gastrointestinal symptoms in 66 percent of patients over a 20-year period. The most commonly reported GI symptoms were abdominal pain (56.1%), nausea (42.3%), constipation (38.6%), heartburn (37.6%), and irritable bowel syndrome-like symptoms (27.5%); however, all gastrointestinal symptoms were more common in the hypermobility subtype. A review of investigations carried out in this cohort showed nearly 40 percent of patients had either hiatus hernia, erosive reflux oesophagitis, or gastritis identified on OGD. Gastrointestinal motility was affected with abnormal gastric emptying in 25 percent and abnormal colonic transit in 30 percent of their patients. The use of medicines was also higher as compared to the general population with 38 percent of their group using PPI on a regular basis and another 23 percent using laxatives [36]. Some studies have shown the prevalence of functional gastrointestinal disorders to be much higher up to 84 percent with nearly half of the studied population reporting irritable bowel syndrome and nearly two-thirds of the population reporting symptoms of gastro-oesophageal reflux [37].

Symptoms of functional gastrointestinal disorders appear to be more prevalent in the hypermobility subtype of Ehlers-Danlos syndrome. A comparison of patients affected by joint hypermobility syndrome/hypermobile Ehlers-Danlos syndrome with matched controls showed that all gastrointestinal symptoms were more common in this group and affected the quality of life adversely [38]. The association between joint hypermobility and functional gastrointestinal is increasingly being recognized although the reasons for that remain unclear [7]. Joint hypermobility appears to be commoner in patients attending gastroenterology clinics for functional disorders than in patients attending for organic disorders [6].

In this study, patients with hypermobility reported a wide range of gastrointestinal symptoms, frequently met the criteria for the diagnosis of functional gastrointestinal disorders, and showed increased use of medications for gastrointestinal symptoms. These appear to be more common in hypermobile Ehlers-Danlos syndrome patients than in Marfan syndrome patients. Both groups scored lower than controls on quality of life indicators; however, hEDS patients had lower mean scores as compared to MS patients.

However, our study does have some limitations. The sample size is small, and although individuals with a known organic gastrointestinal diagnosis were excluded, undiagnosed conditions may have been present. Finally, although it would be expected that all MS patients are under secondary or tertiary care, it is likely that only the severe hEDS would be under rheumatology specialist care. This selection bias might explain the more severe symptoms in this group compared to the MS group.

A greater understanding of functional gastrointestinal symptoms in patients with connective tissue disorders may give further understanding to the aetiology of gastrointestinal symptoms in individuals not formally diagnosed with connective tissue abnormalities and may give insight into the causes of functional gastrointestinal disorders and irritable bowel syndrome.

## Figures and Tables

**Table 1:** Gastrointestinal symptoms in MS/hEDS patients compared with controls.

	Controls	MS/hEDS patients	*P* value (Fisher's exact test)
Number	90	90	
M : F	18 : 72	18 : 72	
Symptoms			
Abdominal pain	25	55	<0.0001^∗∗∗^
Diarrhoea	8	30	<0.0001^∗∗∗^
Constipation	15	49	<0.0001^∗∗∗^
Medicines			
Antisecretory/antacids	17	48	<0.0001^∗∗∗^
Laxatives	13	46	<0.0001^∗∗∗^
Oesophageal disorders			
Functional chest pain	1	6	0.1177
Functional heartburn	11	30	0.0012^∗∗^
Globus	1	3	0.6208
Functional dysphagia	3	13	0.016^∗^
Gastroduodenal disorders			
Functional dyspepsia	7	25	0.0007^∗∗∗^
Belching disorders	4	10	0.016^∗^
Nausea and vomiting disorders	1	4	0.3680
Rumination syndrome	1	3	0.6208
Bowel disorders			
Irritable bowel syndrome	6	21	0.0029^∗∗^
Functional constipation	11	32	0.0004^∗∗∗^
Functional diarrhoea	5	19	0.0036^∗∗^
Functional abdominal bloating/distension	6	23	0.0009^∗∗∗^
Unspecified functional bowel disorder	22	50	<0.0001^∗∗∗^
Centrally mediated abdominal pain syndrome	3	9	0.1324
Functional biliary pain	1	1	1
Anorectal disorders			
Faecal incontinence	1	2	1
Functional anorectal pain	2	4	0.6822

^∗^ *P* < 0.05; ^∗∗^*P* < 0.01; ^∗∗∗^*P* < 0.001.

**Table 2:** Gastrointestinal symptoms in MS patients compared with controls.

	Control	MS	*P* value (Fisher's exact test)
Number	45	45	
M : F	12 : 33	12 : 33	
Symptoms			
Abdominal pain	13	24	0.0315^∗^
Diarrhoea	4	9	0.2297
Constipation	8	16	0.0941
Medicines			
Antisecretory/antacids	9	11	0.8004
Laxatives	6	12	0.1868
Oesophageal disorders			
Functional chest pain	0	2	0.4944
Functional heartburn	5	9	0.3837
Globus	0	1	1
Functional dysphagia	1	4	1
Gastroduodenal disorders			
Functional dyspepsia	3	8	0.1966
Belching disorders	2	3	0.6766
Nausea and vomiting disorders	0	1	1
Rumination syndrome	0	1	—
Bowel disorders			
Irritable bowel syndrome	3	6	0.7136
Functional constipation	5	9	0.3837
Functional diarrhoea	2	7	1
Functional abdominal bloating/distension	3	9	0.1184
Unspecified functional bowel disorder	10	23	0.1760
Centrally mediated abdominal pain syndrome	1	2	0.6164
Functional biliary pain	0	0	
Anorectal disorders			
Faecal incontinence	0	1	
Functional anorectal pain	1	2	1

^∗^ *P* < 0.05; ^∗∗^*P* < 0.01; ^∗∗∗^*P* < 0.001.

**Table 3:** Gastrointestinal symptoms in hEDS patients compared with controls.

	Control	hEDS	*P* value (Fisher's exact test)
Number	45	45	
M : F	6 : 39	6 : 39	
Symptoms			
Abdominal pain	12	31	<0.0001^∗∗∗^
Diarrhoea	4	21	<0.0001^∗∗∗^
Constipation	7	33	<0.0001^∗∗∗^
Medicines			
Antisecretory/antacids	8	37	<0.0001^∗∗∗^
Laxatives	7	34	<0.0001^∗∗∗^
Oesophageal disorders			
Functional chest pain	1	4	0.3607
Functional heartburn	6	21	0.0011^∗∗^
Globus	1	2	1
Functional dysphagia	2	9	0.01^∗^
Gastroduodenal disorders			
Functional dyspepsia	4	17	0.0291^∗^
Belching disorders	2	7	0.2663
Nausea and vomiting disorders	1	3	0.6164
Rumination syndrome	1	2	0.6164
Bowel disorders			
Irritable bowel syndrome	3	15	0.0014^∗∗^
Functional constipation	6	23	0.0002^∗∗^
Functional diarrhoea	3	12	0.0014^∗∗^
Functional abdominal bloating/distension	3	14	0.0059^∗∗^
Unspecified functional bowel disorder	12	27	<0.0001^∗∗∗^
Centrally mediated abdominal pain syndrome	2	7	0.2663
Functional biliary pain	1	1	1
Anorectal disorders			
Faecal incontinence	1	1	1
Functional anorectal pain	1	2	0.6164

^∗^ *P* < 0.05; ^∗∗^*P* < 0.01; ^∗∗∗^*P* < 0.001.

**Table 4:** Quality of life scores in MS patients.

	Controls (mean scores)	MS (mean scores)	Independent sample *t*-test
Physical functioning	100.0	80.0	0.0012^∗∗^
Role limitations due to physical health	100.0	50.0	<0.0001^∗∗∗^
Role limitations due to emotional problems	100.0	100.0	1
Level of energy/fatigue	95.0	45.0	<0.0001^∗∗∗^
Emotional well-being	100.0	68.0	<0.0001^∗∗∗^
Social functioning	87.5	50.0	<0.0001^∗∗∗^
Pain	77.5	67.5	0.1732
General health	100	30.0	<0.0001^∗∗∗^

^∗^ *P* < 0.05; ^∗∗^*P* < 0.01; ^∗∗∗^*P* < 0.001.

**Table 5:** Quality of life scores in hEDS patients.

	Controls (mean scores)	hEDS (mean scores)	Independent sample *t*-test
Physical functioning	100.0	60.0	<0.0001^∗∗∗^
Role limitations due to physical health	100.0	50.0	<0.0001^∗∗∗^
Role limitations due to emotional problems	100.0	50.0	<0.0001^∗∗∗^
Level of energy/fatigue	95.0	30.0	<0.0001^∗∗∗^
Emotional well-being	100.0	50.0	<0.0001^∗∗∗^
Social functioning	87.5	50.0	<0.0001^∗∗∗^
Pain	77.5	50.0	0.0251^∗^
General health	100	45.0	<0.0001^∗∗∗^

^∗^ *P* < 0.05; ^∗∗^*P* < 0.01; ^∗∗∗^*P* < 0.001.

## Data Availability

The questionnaire data used to support the findings of this study are available from the corresponding author upon request.
